# A Tale of Childhood Loss, Conditional Acceptance and a Fear of Abandonment: A Qualitative Study Taking a Narrative Approach to Eating Disorders

**DOI:** 10.1177/10497323231152142

**Published:** 2023-01-18

**Authors:** Roma L. Watterson, Marie Crowe, Jennifer Jordan, Sarah Lovell, Janet D. Carter

**Affiliations:** 1 93831 University of Canterbury, Christchurch, New Zealand; 2 University of Otago, Christchurch, New Zealand

**Keywords:** eating disorders, structural narrative analysis, qualitative research, childhood loss, conditional acceptance

## Abstract

Eating disorders (EDs) are serious mental health illnesses, yet there is a need to better understand the illness experience to improve treatment outcomes. Qualitative research, and narrative approaches in particular, can elicit life stories that allow for the whole illness journey to be explored. This study aimed to explore the experiences of women with a history of an ED, identifying the life events they perceived were relevant to the onset of their ED through to recovery. Interviews were conducted with 18 women with lived experience of an ED. Through structural narrative analysis, an overarching storyline of childhood loss contributing to a belief of conditional acceptance, fear of abandonment and struggle to seek emotional support due to the fear of being a burden was identified. Negative experiences with the health sector were common. These findings have implications for the way medical professionals respond to help seeking and deliver treatment.

Eating disorders (EDs) are serious mental illnesses that can have a range of severe and fatal consequences (Keski-Rahkonen & Mustelin, 2016; Pomeroy & Mitchell, 2002). Treatment outcomes are still limited and dropout from treatment is typically between 20 and 40% (DeJong et al., 2012). A meta-analysis estimated an average dropout rate of 24%, which did not vary significantly across treatment types (Linardon et al., 2018). Furthermore, research indicates that recovery rates from an ED may be less than 50% (Fichter et al., 2017; Silén et al., 2021; Steinhausen & Weber, 2009), with a recent study reporting rates as low as 29% (Eielsen et al., 2021). The need to better understand these disorders to improve treatment outcomes is evident.

Research has demonstrated the importance of acknowledging the patient’s view to improve treatment efficacy. For instance, it has been suggested that, within the mental health sector, a patient’s beliefs about their illness will determine which treatments they think will be effective, how they respond and their adherence to treatment plans (Petrie et al., 2008). Patients with EDs have been found to be more likely to drop out of treatment if they do not perceive it to be credible (Jordan et al., 2017), demonstrating the importance of considering patient views.

Qualitative research from the perspective of those with lived experience of an ED provides an important contribution to existing knowledge about these complex illnesses. Through qualitative studies, we can gain a more in-depth understanding of a phenomenon and the meanings participants attach to their experiences in the context of their social world (Ritchie & Lewis, 2003). Previous qualitative research has found that people with lived experience of an ED identified the main causes of their ED as low self-esteem (Dignon et al., 2006; Gulliksen et al., 2017; Nilsson et al., 2007; Redenbach & Lawler, 2003; Tozzi et al., 2002), a need to feel in control (Button & Warren, 2001; Fox & Diab, 2015; Rossotto et al., 1996; Tozzi et al., 2002), trauma and abuse (Dignon et al., 2006; Gulliksen et al., 2017; Lacey et al., 1986; Nilsson et al., 2007; Tozzi et al., 2002), perfectionism (D’Abundo & Chally, 2004; Gulliksen et al., 2017; Nilsson et al., 2007; Redenbach & Lawler, 2003) and body dissatisfaction (Dignon et al., 2006; Gulliksen et al., 2017; Nilsson et al., 2007). When asked why their ED was maintained, participants in qualitative studies have attributed this to their struggle to cope with negative emotions and the belief that their ED enabled them to manage this struggle (Fox & Diab, 2015; Gulliksen et al., 2017; Lacey et al., 1986; Rossotto et al., 1996).

Narrative approaches preserve the social context surrounding each participants’ account, allowing their story to unfold, including the order of events, how different events are believed to be interconnected, how perceptions can change over time and the meaning behind experiences (Riessman, 2008; Stephens, 2011). Within the context of EDs, narrative research has the potential to enable greater understanding of the whole illness experience from onset to recovery. Existing qualitative research investigating EDs has mainly focused on causal and maintenance factors separately to treatment and recovery, rather than considering the whole journey.

Of the few narrative studies on EDs identified, two focused on the experiences of athletes using a case study approach; one was a single case study (Papathomas et al., 2015) and the other compared the experiences of one male and one female athlete (Busanich et al., 2014). Another study was restricted to experiences with specialist ED services and found a lack of compassion and collaboration was resulting in a revolving door experience of treatment (Joyce et al., 2019). There are no narrative studies exploring an individual’s journey of an ED.

The aim of the present study was to explore the experiences of a sample of women with anorexia nervosa (AN), bulimia nervosa (BN) and binge eating disorder (BED), identifying the life events they perceived were relevant to the onset of their ED through to their recovery or present day (if not recovered). An additional aim was to identify any common plots in the narratives of these women that may have implications for improving treatment efficacy.

## Method

### Qualitative Approach

Despite the benefits of narrative research, particularly the ability to elicit personal life stories, this approach has been under-utilised with people who have lived experience of an ED. A narrative approach was taken to the present qualitative study, enabling participants to share their story of their ED, including the events they perceived to be relevant and why, and how events may be interconnected. Whilst accepting that a participant’s recollection is not a universal truth, and one person’s experience cannot be applied to everyone, this approach provided an insight into how the participants’ earlier experiences shaped their later responses to treatment, and how the patient’s history may increase treatment efficacy.

This qualitative approach is also aligned with the ontological position from which the research was carried out. The study was conducted from a critical realist position, which assumes that although there is a world independent of human existence, our social experiences determine our knowledge and shape our perception of reality (Danermark et al., 2005). Danermark et al. (2005) stated that researchers need to know the meaning and significance people assign to their actions in order to understand those actions. The narrative approach taken here allowed participants to not only describe life events but their views on why those events may have influenced subsequent behaviours and the motivations behind their actions.

The critical realist position assumes that humans construct their own meaning of the world from their social experiences; therefore, it stands to reason that researchers are influenced by their own experiences which, in turn, shape the research (Clarke & Braun, 2018; Snape & Spencer, 2003). The lead researcher (who conducted all interviews and the main analysis) would be considered as an ‘outsider’, as they had not experienced an ED, but sought to build authentic dialogue with participants enhanced by narrow social distance (shared gender, ethnicity; Reid et al., 2010). Reflexivity was a relevant tool used here, which refers to the researcher being cognisant of their own views, in order to recognise and take into account the influence of power relations on the research findings (Berger, 2015; Reid et al., 2010). Considering the rationale behind decisions was key to the reflexive process throughout design, data collection, data analysis and the presentation of findings. The researcher also reflected back on each interview, to determine how it might have been approached differently and any learnings for the next interview. In addition to reflexivity, this iterative approach demonstrated researcher responsiveness or flexibility, a concept Morse et al. (2002) proposed as central to verification for qualitative research. A further step taken to enhance the richness of the analysis was obtaining peer agreement on the key events, themes and storylines identified (Morrow & Smith, 2000).

### Participants

Participants in the present study were 18 women with lived experience of an ED; 7 with AN, 7 with BN and 4 with BED. Participants ranged in age from 16 to 51 years and were mainly New Zealand (NZ) European (*n* = 16). Therefore, when considering the findings, the gender and ethnicity of participants should be acknowledged. Half of the participants considered themselves to be recovered (*n* = 9). Participant information is presented in Table 1. All the women had previously completed an online survey as part of the Costs of Eating Disorders in New Zealand study (the COSTS study; Jordan, 2017) and provided their contact details if they were willing to be contacted about further research. A total of 35 participants from the COSTS study met this criteria and were contacted, making a response rate of 51.4%. Pseudonyms were assigned to participants to protect anonymity.

**Table 1.** Diagnostic Subtype, Perceived Recovery Status and Age of Each Participant, Identified Using Their Assigned Pseudonym.

Pseudonym	Diagnostic Subtype	Recovery Status	Age Band (Years)
Daniella	AN	Non-recovered	31–40
Pippa	AN	Non-recovered	16–20
Tracey	AN	Non-recovered	16–20
Helen	AN	Recovered	21–30
Maddie	AN	Non-recovered	16–20
Jan	BN	Recovered	41–50
Anika	AN	Recovered	16–20
Beth	BN	Recovered	41–50
Kelly	BED	Non-recovered	41–50
Zoe	AN	Recovered	21–30
Kay	BN	Recovered	41–50
Alice	BED	Non-recovered	41–50
Millie	BN	Non-recovered	21–30
Christina	BN	Recovered	51–60
Andrea	BED	Non-recovered	31–40
Miranda	BN	Non-recovered	21–30
Linda	BN	Recovered	41–50
Catherine	BED	Recovered	41–50

Recruitment for the COSTS study was broad, including promotion through the Eating Disorders Association of New Zealand (EDANZ) website and mailing list, posts on university and community group social media pages, and hard copy posters displayed in central community locations such as libraries, supermarkets and community centres. Eligibility criteria for the present study included female, aged 16 years or over, and with a diagnosis or probable diagnosis of AN, atypical AN, BN, subthreshold BN, BED or subthreshold BED. Gender, current age and diagnosis were all self-reported through the online survey. Eligible participants who provided contact information through the COSTS study were invited to participate. All participants provided written informed consent to participate in the interview. Ethics approval for the study was provided by the Health and Disability Ethics Committee (HDEC) (ref: 16/NTB/189/AM01).

### Data Collection

A semi-structured interview was conducted with each participant, consisting of open questions phrased to encourage participants to share their own story. A small number of questions were relevant only to the COSTS study. High level interview questions pertaining to the current study are provided in Table 2. Prior to the interview, participants were provided with an information sheet explaining the purpose of the study and the types of questions they would be asked. Participants were asked ‘can you talk me through your experience of your eating disorder from the beginning, how did it start and how did it go on from there?’. If not spontaneously provided, participants were also asked what they thought caused or contributed towards the start of their ED, why they thought it was maintained, their experience of treatment and their experience of recovery. If topics or events of a particularly sensitive nature were mentioned early in the interview, they were revisited by the researcher once a greater level of rapport had been built. All interviews were audio-recorded with the participant’s consent.

**Table 2.** Semi-Structured Interview Questions Pertaining to the Current Study.

Can you talk me through your experience with your eating disorder from the beginning? How did it start? How did things go on from there?
What do you think caused or contributed towards the start of the eating disorder for you? Why do you think it was maintained?
o If not mentioned ask (including specific examples) about:
⁃ Personality traits
⁃ Self-esteem (feelings towards self and how they saw self)
⁃ Experiences of stress or trauma
⁃ Coping
⁃ Control
Please tell me about your experience of treatment.
o If not mentioned ask about:
⁃ Difficulties and positive aspects
⁃ What would you look for in an ideal eating disorder treatment? What improvements could be made?
⁃ Relationships with professionals
Can you talk me through your experience of recovery?
o What do you think were the most important factors in helping you to improve or recover from the eating disorder?
o Who or what, if anything, was helpful in addressing any of the causes or maintenance factors already discussed?
⁃ Why or how did it/they help?

Interview data was collected until data saturation was reached. While there is no single accepted process for determining data saturation, here the term refers to the point at which new data did not further develop the findings (Francis et al., 2010). This occurred when the analysis of a new interview did not alter the overarching narrative and key plot points previously identified.

### Data Analyses

Audio files were transcribed by the researcher for analysis. This process enabled the researcher to become immersed in the data. Next, each narrative was summarised to provide an overview of the participant’s experience and the events they highlighted as key to their story. Structural narrative analysis was then carried out on each summary, using the Labovian approach, which understands personal narratives to be a representation of a series of past events in the form of a story. Labov’s (1972) model of the structure of personal narrative separates narratives into six main elements from which the story is constructed:

• Abstract – summarising the purpose of the narrative (this was not included in the present study as participants had been asked to focus on a certain part of their life);
• Orientation – information about the time, place and situation to give context;
• Complicating action – the key events of what happened, usually involving a crisis and a turning point, the main body of the narrative;
• Evaluation – thoughts and feelings about what happened, the emotion of the narrative;
• Resolution – the outcome or how the action ended; and
• Coda – the end which brings the narrative back to the present.

The Labovian approach has been proposed to be a rigorous method for analysing personal narratives, particularly as the systematic application of the model allows for comparison across narratives (Patterson, 2013). The narrative summaries for each element were compared to identify common themes and events that formed that element. Then, when considered together and in order, the key themes for each element constituted the overarching storyline.

## Results

### Overview

The 18 women who shared the story of their ED for this research had individual experiences but there were also similarities, regardless of ED diagnosis. This was particularly evident in the structure of their narratives and the core themes that constituted each element. All narratives were full of emotions and consisted of multiple struggles; there was a clear sense that their ED had dominated (or was still affecting) a large part of their life. From the core themes, an overarching narrative was identified.

**Figure 1.**
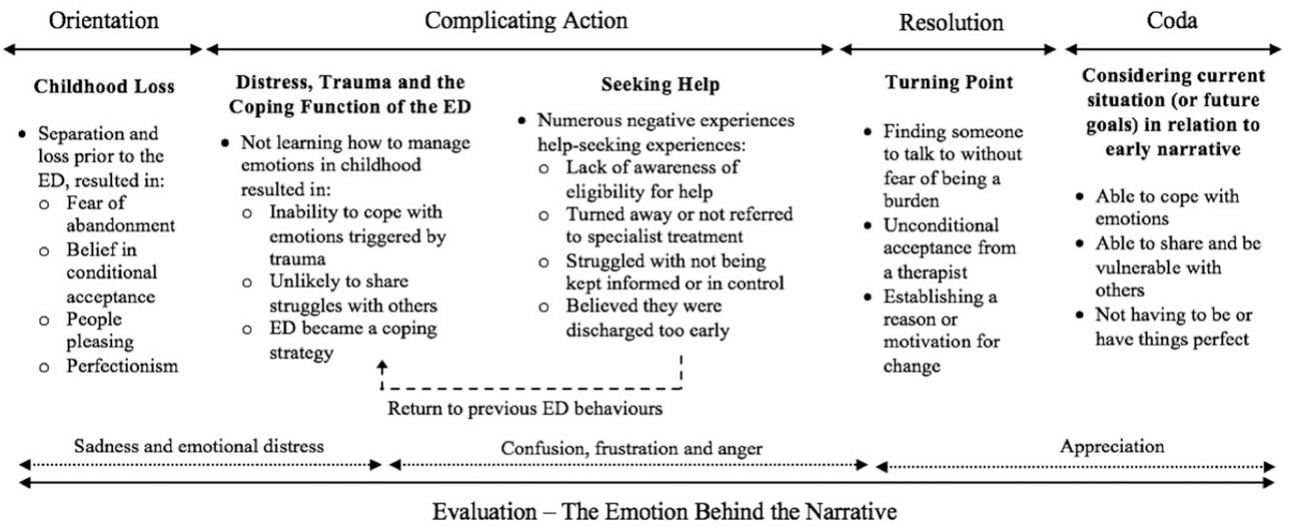
An illustration of the overarching narrative, broken down using Labov’s (1972) model of the structure of personal narrative, detailing the key themes that constitute each element of the narrative.

The overarching narrative began with early childhood recollections, involving separation and loss, prior to the onset of the ED; frequently, these recollections also involved early memories of food. Participants explained that their childhood contributed to a struggle to manage distress, meaning an inability to cope with later trauma. The ED then became a coping strategy and a central character in the narrative. Attempts to reach out for help were often rejected, and often treatment was considered inadequate. This barrier to recovery constituted the core complicating action in the overarching narrative. The inability to get the help needed allowed the ED to become deeply ingrained, and fuelled frustration and anger at the health sector. For those participants whose narrative reached some form of resolution, this either came as a result of eventually finding a therapist with whom they could share or an event that triggered a re-valuing of life. Notwithstanding the unique experience of each individual, the overarching narrative structure and themes identified are illustrated in Figure 1 and are considered in detail below.

### Orientation

#### Childhood Loss

Participants began their narratives by recalling their lives before their ED, with a focus on their character and events they believed may have had an influence. There was a common theme of separation and loss, often accompanied by the presence of food. Two thirds of the participants described the breakup of their family, being separated from their family (such as foster care) or loss of a family member at a young age. Many of these participants spoke of subsequent loneliness and compensating with food:

[Sister] leaving home was a real wrench. I think it was done with good intentions all round but ... it felt like they didn’t consider the impact it would have on me... I think I was lonely, I think I just missed her. She understood me, she knew me completely, she accepted me and suddenly I didn’t have that anymore. So, yeah, it was comfort eating for sure. [Kelly]

I’d never really been away from my dad and we were really close, you know, like a proper daddy’s little girl sort of relationship, and I really do remember missing him, but also food being around a lot... Maybe in compensation for the fact that we were here and this sad thing was happening, the family was split up. [Linda]

The loss was often internalised as a form of abandonment, an experience that those participants believed caused low self-worth or a lack of unconditional self-acceptance, a fear of future abandonment and a subsequent need to please others to avoid rejection:

So I had these two tapes that run in my head. The first would be ‘you’re not good enough’ and that sort of sat with ‘your mum’s left you … as a 3-year old’ that sort of you’re not good enough. And then the second one would be ‘you must please everybody and make sure everybody is happy so that they don’t leave you’. [Andrea]

A lack of unconditional self-acceptance, the belief that others have high standards of conditional acceptance, being ‘scared of being rejected’ [Christina] and fearing abandonment if they failed to meet standards was a common storyline:

I don’t think as a small child I truly believed that they loved me unconditionally, which I guess always made me feel I could be abandoned. [Linda]

Just under half of the participants spoke of putting the needs of others before their own, and fearing upsetting others or letting people down. Prioritising the needs and wellbeing of others over their own, with the goal of gaining acceptance or avoiding abandonment, meant these participants were unlikely to share their own struggles. Additionally, around a third of the women referred to themselves as being a perfectionist and some of these women described striving to be perfect as a way of seeking acceptance from others. Millie, for example, said: ‘I always felt like whatever I did it wasn’t good enough for him… I was always like “I have to be perfect so that Dad cares”… I was trying to get these really good grades to make him proud’.

### Complicating Action

#### Distress, Trauma and the Coping Function of the ED

There was a lack of certainty over exactly when the ED began, as behaviours gradually escalated over time. The overarching narrative did, however, feature some form of distress or trauma before or during the initial stages of the change in eating patterns. Although only one participant used the term ‘trauma’ [Kelly], and one a ‘traumatic’ experience [Jan], where phrases were considered to show a similarly high emotional impact including ‘that really upset me’ [Miranda], ‘made me really unhappy’ [Anika] and ‘that’s when my world came crumbling down’ [Andrea], these events were categorised as trauma. In addition to childhood loss, these experiences of trauma consisted of sexual abuse (approximately a quarter of participants), bereavement (around a sixth of participants), bullying and having a disabling accident (each relevant to a small number of participants). Additionally, participants frequently spoke of events that caused distress, which mainly focused on the end of an intimate relationship and struggles or pressures at school. Early childhood loss was described as influencing an inability to cope with the emotions that were then triggered by later trauma and/or distressing life events. Early family disruption and subsequent loneliness and isolation, was a barrier to learning how to cope with emotions, as there was nobody to learn from or share with:

I didn’t know how to, how to, er, deal with loss. Erm, my brother died many, many years ago and I never got taught by my parents how to. [Jan]

Participants therefore started ‘not telling anything to anyone’ [Jan] and resulting, for some, in being ‘quite shut off emotionally’ [Kelly].

It was at this point, struggling to cope with distress and other negative emotions, that the narrative turned to the relationship with food. Almost every participant described a realisation that, whether it was through restriction, binge eating or purging, food could provide a way to feel better. Predominantly this was achieved through giving a sense of comfort or control:

I think m-maybe partially because when I was quite little I was really anxious … I would use food as a comfort because, it hadn’t- it hadn’t really been taught to me, but I guess it worked. [Miranda]

I stopped eating. I just stopped and it was control over my body and control over the situation. [Beth]

Most participants recalled how their eating behaviours then became an intentional way to cope. It was at this juncture that the ED was introduced to the narrative, as something to turn to when faced with any kind of negative emotions. For a couple of women, it was not only negative but positive emotions too, with one participant saying ‘it’s really emotional stress, if I was angry, sad, so extreme emotions, even if I was really happy’ [Kay], and another recounting a time she met an old friend which left her ‘feeling amazing’ and those feelings triggered her to binge eat.

For several participants, their ED was portrayed as a distinct character, with its own voice and behaviours. These participants described the voice as hijacking recovery by encouraging unhealthy behaviours with food:

The eating disorder tells you that when you get to your high weight, you’re going to feel even worse about yourself, you’re going to feel so sad. [Tracey]

You get into this mind frame of, I don’t even think its yourself anymore, it’s just this other kind of thing or this other person that, erm, praises you when you encourage those harmful behaviours, so there are moments when your emotions, erm, are quite elated. [Helen]

In the aforementioned quote, Helen explains that the ED voice made her feel better by praising her. Other participants described using their ED to ‘push the feelings down’ [Catherine], ‘block the bad emotions’ [Kelly] and ‘block out what was actually going on’ [Zoe]. Thus, a central component of the overarching narrative was the conceptualisation of the ED as a coping strategy that served a positive function (e.g. emotion regulation) rather than as an illness.

#### Seeking Help

As the ED was generally perceived as having a positive function at this point in the narrative, treatment was mainly first approached as a result of someone else. Some were taken to a medical professional by a family member and others were introduced by someone else to the possibility that their behaviours may not be a normal way of coping. Whilst seeking help could be expected to signal the beginning of a resolution, instead, a plot twist occurred. Rather than a smooth flow into treatment and through to recovery, a series of barriers were encountered.

Firstly, simply accessing treatment was a challenge for some, especially those who were not underweight and thought they ‘don’t look sick’ [Miranda], weren’t ‘sick enough to get help’ [Helen], or would be judged: ‘it’s that stigma…some who’s wobbling down the street really overweight [it’s] just lack of self-control’ [Linda]. Several others were turned away from treatment or their doctor failed to refer them to a specialist. This left Millie feeling like ‘it’s not an actual problem… like it’s not validated, it’s not a real thing’ and ‘scared to talk about it’.

The majority of participants who were taken into inpatient treatment recalled a lack of understanding over what was happening to them and why, as ‘they don’t explain anything, you’re kind of in the dark with everything all the time’ [Tracey]. Not being kept informed or involved in decisions enough meant they did not feel in control:

I didn’t feel like I was in charge of my own recovery or I was in charge of getting better... it was just really pointless. [Maddie]

These participants also felt they were discharged too early:

They only keep you until you’re kind of at a sort of stable weight, like not even weight but stable medical status and then they let you go... they didn’t think at all about what was maybe going on inside my head. [Pippa]

Regardless of the individual treatment path or experience, the narrative circled back to the same situation as before the initial interaction with a medical professional. The ED continued and some believed they must be well or they would not have been turned away or discharged.

During this part of the narrative, timelines jumbled slightly as participants struggled to recall the order of their experiences due to repeatedly moving in and out of treatment. It was mainly due to self-determination and desperation that participants persevered with different treatments or therapists until they found someone who helped, with one participant describing herself as ‘really lucky I am pig headed and I’m determined’ [Andrea]. Another participant recalled:

I remember I came out of one session literally screaming, crying, I was like ‘please help me’ … I just needed help and no-one was giving it to me…[I’d been] to see the doctor, to see family treatment, and then to individual sessions… We didn’t have a connection so I couldn’t speak to her about anything… Then I was like ‘we’re going to go private’… and she was really good and she really helped me. [Tracey]

This quote epitomises the treatment journey of the majority of participants; trying a range of treatments, desperation to find a treatment that worked and the importance of the right therapist.

### Resolution – Turning Points

A small number of participants were still struggling with their ED. The majority, however, had experienced a turning point that enabled them to move toward recovery. Being able to talk openly to someone ‘and not be worried about emotional consequences on other people’ [Helen] or being ‘a burden to them’ [Millie] was an important step in working toward a resolution; overcoming their drive to prioritise the wellbeing of others. Therapists who demonstrated unconditional acceptance reduced fears of abandonment and rejection, which encouraged talking openly:

She was perhaps my initial realisation, albeit therapeutically, [that someone could] love me unconditionally, because I showed her some pretty dark sides ... of myself and she didn’t go anywhere. [Linda]

Meeting a therapist who understood and met individual needs was vital because successful treatment ‘varies from person to person’ [Maddie] but identifying ‘reasons for wanting to solve it’ [Alice] was a central part of reaching the resolution. Motivation for change often stemmed from ‘looking forward’ [Kay] and was sometimes triggered by factors outside of treatment, such as a sudden realisation of mortality from a health scare, having children or grandchildren, moving to a new area or starting a new job: ‘I’m definitely more aware and I think possibly cancer has contributed to that… I’ve got better reasons behind wanting to solve it now, cancer and a child… It’s a pretty big driving force’ [Alice].

### Evaluation – The Emotion Behind the Narrative

Despite the personal and intimate emotions, there were parallels across participants in the way they reflected upon their experience. There was a high level of sadness when reflecting on childhood experiences and low self-worth. A small number of participants were moved to tears when speaking of how negatively they had viewed themselves and the impact that had on their life, such as the experiences they missed out on. One participant, for example, emotionally recalled a moment when ‘it occurred to me … that I can’t die because I haven’t really started living yet’ [Christina].

As the narrative moved on to first experiences with health professionals and treatment, the tone appeared to change to one of confusion and frustration, often focused on the belief that they received a lack of psychological support, explanation or individualised treatment:

I just found it so frustrating to be told like first we have to deal with what you’re struggling with the most without the psychological support, which is really what it is entirely. It’s entirely psychological, so it makes no sense. [Helen]

At this stage, there was also a level of anger expressed, demonstrated through profane language and words such as ‘ballistic’, [Daniella] ‘angry’ [Tracey] and ‘shocking’ [Helen].

The overarching narrative ended, however, on a more positive note, focusing on improvements and an expression of gratitude. Although the interview schedule directed the conversation towards improvement and recovery as the final discussion point, this appreciation for how far they had come was not prompted.

### Coda – Bringing the Narrative to a Close

Despite each participant being asked the same final question regarding their experience of recovery (see Table 2), there was a slight variation in the way participants finished their stories depending on their perceived stage of recovery. Those who saw themselves as fully or mostly recovered reflected back on the struggles they identified early in their narrative and explained why they are no longer an issue, bringing the narrative full circle, such as being able to ‘now be vulnerable with people’ [Linda], ‘internally tolerate a lot more [emotion]’ [Linda] and ‘accepting that things don’t have to be perfect’ [Kay]. Alternatively, participants who considered themselves to still have a long way to go on their journey looked toward their future and their plan to ‘hopefully break the cycle somewhere along the line’ [Andrea].

## Discussion

All 18 women gave detailed accounts of their individual experiences of their ED. The impact their illness had had on their lives was evident. Although each narrative was unique, an overarching storyline was identified. The main storyline identified across the present sample centred around loss and trauma; catalysts for the belief that acceptance was conditional and a fear of abandonment if they failed to meet those conditions. Participants articulated many connections between past and future events. For instance, separation from loved ones at a young age appeared to motivate behaviours that sought to limit the risk of future abandonment, including not wanting to burden health professionals with their ED. These connections demonstrate the importance of considering the journey as a whole and the benefit of taking a narrative approach.

### Overarching Storyline – Conditional Acceptance and Fear of Abandonment

In line with the present findings, fear of abandonment as a result of childhood experiences, such as parental divorce (Guinart & Grau, 2014), on-going parental conflict (Hayashi & Strickland, 1998) or bereavement (Black, 1996), has been established in the literature. For the participants in the current study, early separation from a loved one appeared to have resulted in a fear of future abandonment and a subsequent prioritisation of the needs of others, to the detriment of their own wellbeing, to negate the perceived risk of future rejection. Research has found an association between the presence of maladaptive schemas, including self-sacrifice and abandonment, and EDs (Jones et al, 2007; Waller, 2003), and promising results for the use of schema-focused therapies in EDs (McIntosh et al., 2016; Pugh, 2015). Similar to the current findings and aligning with the high prevalence of EDs in women, it has been proposed that AN may be influenced by the gender stereotype that women should be self-denying and achieve emotional fulfilment though the nurturing of others, leading to a preoccupation with ‘being for others’ rather than themselves (White, 1986). Yet, the link between childhood loss, self-sacrifice, fear of abandonment and EDs has not been thoroughly investigated.

Fear of abandonment is a recognised feature of attachment insecurity, and research into attachment styles did find that attachment insecurity was higher in individuals with an ED and may have been related to treatment dropout and poor outcomes (Tasca & Balfour, 2014). Children with an insecure attachment style have also been found to have less understanding of negative emotions and be less likely to share their experiences of negative emotions with their parents, which may put them at risk of emotion regulation difficulties (Waters et al., 2010). The link between struggles to regulate emotions and EDs has been well established (e.g. Brockmeyer et al., 2014; Harrison et al., 2010) and was evident in the narratives of the women in the present study. Furthermore, in their work detailing the use of emotion-focused therapy for individuals with EDs, Dolhanty and Greenberg (2007) proposed that this struggle stems from a lack of or problematic response to their emotional needs from a young age. The authors explained that these difficulties can be intensified by emotional events such as loss and abuse; a claim that appears to be supported by the findings from this present study.

Another relevant factor may be self-esteem. Although participants in the present study did not explicitly discuss low self-esteem as part of their fear of abandonment, they spoke of not feeling good enough for the acceptance of others and this was seen as a reason why someone may leave them. Low self-esteem is a well-established factor in EDs (e.g. Fairburn et al., 2003; Polivy & Herman, 2002); however, the role of conditional acceptance in the onset, maintenance and treatment of EDs is under researched. Low unconditional self-acceptance has been associated with depression and, consistent with the current research, perfectionism (Flett et al., 2003; Scott, 2007). Through the use of case studies, Flett and Hewitt (2002) demonstrated that perfectionists not only frequently have low self-acceptance, but suffer a preoccupation with trying to gain approval of others. Similarly, the findings in the present study suggested that participants sought to gain approval or acceptance of others, but this need for approval may stem from separation or loss as well as perfectionism. It appeared that, in the present sample, perfectionism may have developed as a way of striving to achieve conditional acceptance and, in doing so, reduce the risk of rejection or abandonment. An alternative possibility, however, could be that participants sought to find a way to justify their perfectionist tendencies within their account, although this theory was not strongly supported by the data here. Whilst a link between perfectionism and EDs has been well established (e.g. Bardone-Cone et al., 2007; Egan et al., 2011; Wade et al., 2016), further research may be required to better understand any relationship between perfectionism and childhood loss.

Qualitative research from the perspective of women with lived experience of an ED that address fear of abandonment in EDs and ED treatment is extremely limited. Leavy et al. (2011) interviewed patients who had failed to attend appointments at an ED clinic and found, similar to the present study, that several had experienced past separation, displayed fears of abandonment and had previously had negative experiences of treatment. The authors concluded that these factors culminated in a difficulty building trusting relationships. The need to build trust to establish a good therapeutic relationship has been well established (e.g. Button & Warren, 2001; Fox & Diab, 2015). Findings from the present study expand upon existing research and suggest that, for some women, understanding fear of abandonment and perceptions surrounding conditional acceptance are likely to be important for building and maintaining a good therapeutic relationship. Further, participants in the current study described a fear of being a burden or having a negative emotional impact on people, including professionals. Other recent research found concerns about worrying others was a strong barrier to help seeking for people with ED symptoms (Ali et al., 2020). For the women in the present study, concerns about impacting others was identified as a barrier to engaging within treatment, and overcoming these concerns was a key turning point within therapy. Being cognisant of the fear of having an emotional impact on their therapist therefore appeared to be an important aspect of an effective therapeutic relationship for the sample of women in this study.

### Plot Complication – Barriers to Treatment and Recovery

In addition to needing to find the right therapist and therapeutic environment, participants faced an alarming number of other barriers to treatment engagement. Many struggled to differentiate between negative experiences, and recollections of multiple experiences were frequently blurred together, demonstrating that a negative experience when attempting to seek help was not an isolated event. It was in fact very common.

Stigma and shame were found to be the most frequently identified barriers to help seeking in a review, with the belief that society thinks EDs are a lifestyle choice not a real illness, fostering shame and embarrassment (Ali et al., 2017). In the present study, however, the stigma discussed was associated with being overweight and how this could be a barrier, alongside people being unaware you do not have to be severely underweight to have an ED or be entitled to treatment; demonstrating the impact of low health literacy on help seeking. Consistent with Ali et al. (2017), several participants in the present study did describe embarrassment over their struggles and a fear that they would not be taken seriously as barriers to help seeking. Other people played a large role in prompting them to seek treatment. Evans et al. (2011) found women with EDs were reluctant to raise the topic of eating but were frustrated it was not brought up when seeking help for weight-related issues. When considered alongside the research by Evans et al. (2011), findings from the current study suggest that medical professionals should be trained to screen for an ED and to start a dialogue that invites patients to open up, rather than waiting for the patient to initiate the conversation.

It appears that limited knowledge of EDs within the health sector can be an issue, including the failure to identify an ED. Some participants recalled inappropriate responses from health professionals when they broached the subject, and were not referred to specialist treatment, implying they encountered a lack of knowledge and understanding. It has been suggested that limited training and resources in the health sector may be a contributing factor to limited understanding in health professionals (Reid et al., 2010; Surgenor & Maguire, 2013). Moreover, research has demonstrated the importance of early intervention in EDs for improving treatment outcomes (Allen et al., 2020), particularly for AN (Treasure et al., 2015). The recently developed ‘first episode rapid early intervention for eating disorders (FREED)’ service model, which includes contact being established within 48 hr of a referral, with a focus on active outreach and engagement as well as early symptom change, has been shown to be effective at increasing sustained weight gain and reducing hospital readmissions (Allen et al., 2020). When considered alongside the embarrassment, denial and uncertainty over eligibility for treatment, that were prominent in the narratives in the present study, it appears crucial that EDs are identified as soon as possible so the person can receive an urgent intervention.

Several participants who entered inpatient treatment felt that they were discharged too early, suggesting, as proposed by Offord et al. (2006), that a greater reintegration programme and higher level of structured support post-discharge may have been beneficial. Previous research has found that patients, nurses and parents had differing opinions on discharge readiness and needs (Turrell et al., 2005). Turrell et al. (2005) found that patients believed they would need intensive support from friends and family, but parents showed denial over the severity of their child’s illness and the importance of providing support to their child. While participants in the present study did not explicitly state their parents provided insufficient support, the findings in combination with existing research highlight the importance of considering the patients view of their readiness and the strength of their support network when arranging discharge.

### The ED as a Distinct Character

Aligning with those participants who portrayed their ED as a distinct character in their narrative, the conceptualisation of the ED as a separate entity is often utilised in treatment to help patients distance themselves from their illness. The concept of an ‘anorexic voice’ is also something that has been discussed in the literature. It has been estimated that over 90% of people with an ED experience a critical inner voice (Noordenbos et al., 2014); however, research into the function this voice serves is limited. From a small qualitative study, Tierney and Fox (2010) posited that the ‘anorexic voice’ began as something that facilitated emotional avoidance and gradually became more critical, so it was hard to let go as it was initially valued. In one of the few studies into the nature of the voice experience, Pugh and Waller (2017) reported that a stronger voice was associated with a longer illness duration and concluded that increased understanding of the anorexic voice and how to address it could be important for increasing outcomes in severe and enduring AN. Yet, a review concluded that the experience of an anorexic voice and the implications it has for treatment are not well understood (Pugh, 2016). The present research suggests that the role of an internal voice could extend beyond AN to other ED diagnoses.

Research into the existence of an ‘eating disorder voice’ rather than solely an ‘anorexic voice’ is limited. Consistent with traumatic-dissociative theories of voice-hearing, the power of an ‘eating disorder voice’ was found to be associated with childhood emotional abuse; a relationship partly mediated by dissociation (Pugh et al., 2018). Pugh et al. (2018) concluded that voices may derive from an internalisation of experiences of bullying, or rejection, as the voice is often similarly critical. It has also been posited that an internal voice, even if critical, may reduce feelings of loneliness (Tierney & Fox, 2010), which may explain why it can occur following isolating experiences. The present study suggests childhood loss or separation may be internalised in a similar way. The belief of not being good enough and prioritising the needs of others to reduce the risk of being abandoned, may be heightened by an internal critical voice, or ‘eating disorder voice’. The potential role of this voice across all ED diagnoses, but particularly BN and BED, requires further research.

### Implications and Future Directions

Notwithstanding the inability to generalise the experiences of the women in this study to all who have experienced an ED, the findings have several important implications for future research and clinical practice. The study found conditional acceptance, a fear of abandonment and prioritising the needs of others to be important and inter-related factors across the narratives of these participants. Further research is needed into the role of these factors in EDs, including implications for treatment. For example, it may be beneficial to provide greater attention to identifying abandonment and self-sacrifice schemas in treatment and increasing the focus on these when they are present.

Recognising the concerns people with an ED may have about their impact on others could have implications for the way clinicians deliver treatment, including having some control over their treatment and keeping them informed of what is happening. Acknowledging those aspects of treatment participants highlighted as helpful, namely, therapists who showed unconditional acceptance, treated them as an individual and did not show signs of emotional impact, also has implications for health professionals in building a strong therapeutic relationship. A summary of the key implications for health professionals identified from the findings is set out in Table 3; taking these findings into consideration when raising awareness and delivering therapy may encourage people to seek help and reduce dropout from treatment. Looking to increase help seeking for EDs is vital. It is evident that there are still some health professionals who are not responding appropriately to people who disclose concerns about their eating habits. Research into the current knowledge levels of professionals, particularly those in gatekeeper roles to specialist treatment, may help to understand the extent of the issue.

**Table 3.** A Table Summarising the Key Findings Alongside the Implications for Health Professionals.

Key Findings From The Study	Implications for Health Professionals
Participants appeared to demonstrate a fear of abandonment and a tendency to prioritise the needs of others to the detriment of their own wellbeing	• Identify abandonment and self-sacrifice schemas, and include attention to these schemas in therapy when relevant • Demonstrating unconditional acceptance may help to build a strong therapeutic relationship
Many participants feared being an emotional burden to others and found it helpful when health professionals did not appear to be emotionally affected when they shared their struggles	• Emphasis should be placed on providing a regulated response to emotional disclosures so as not to reinforce concerns around emotional burdening
Several participants described struggling with not having any control over their treatment and not being kept informed of what was happening	• The role of control in EDs should be considered during treatment, for example, involving the individual in decisions and explaining what is going to happen and why it may help them to feel some control
Most participants said it was helpful to be treated as an individual	• Individual treatment plans that consider each person’s unique history and circumstances are encouraged. If standard treatment protocols are in place, demonstrating that each person is seen as an individual is important
Most participants who were not underweight during their ED experienced this as a barrier to help seeking, either through lack of awareness that they were entitled to help or a stigma associated with being overweight	• Increase education for health professionals (and the public) around ED subtypes, how to encourage disclosure and how to respond to it • Raise public awareness of who can access ED services, especially for BN and BED

## Conclusion

This was a qualitative study with a sample of women who spoke of a range of distressing life events and how they believed these events were inter-related with their lived experience of an ED. Through this research, an overarching narrative was identified from their stories. Suffering childhood separation or loss, not learning to cope with emotions, prioritising the needs of others, striving to be perfect to achieve conditional acceptance and fearing letting others down or abandonment were key experiences within that narrative. Therefore, receiving help to cope with emotions, not feeling like an emotional burden and being allowed time to build rapport with therapists were helpful for treatment and recovery. These findings provide clear evidence for the importance of acknowledging and understanding how each client’s life experiences have shaped them as a person, to provide appropriate support in the most effective way for that individual.
